# Carbon nanotube filled with magnetic iron oxide and modified with polyamidoamine dendrimers for immobilizing lipase toward application in biodiesel production

**DOI:** 10.1038/srep45643

**Published:** 2017-03-30

**Authors:** Yanli Fan, Feng Su, Kai Li, Caixia Ke, Yunjun Yan

**Affiliations:** 1 Key Laboratory of Molecular Biophysics of the Ministry of Education, College of Life Science and Technology, Huazhong University of Science and Technology, Wuhan, 430074, P. R.China

## Abstract

Superparamagnetic multi-walled carbon nanotubes (mMWCNTs) were prepared by filling multi-walled carbon nanotubes (MWCNTs) with iron oxide, and further modified by linking polyamidoamine (PAMAM) dendrimers (mMWCNTs-PAMAM) on the surface. Then, mMWCNTs-PAMAM was employed as the carrier and successfully immobilized *Burkholderia cepacia* lipase (BCL) via a covalent method (BCL-mMWCNTs-G3). The maximum activity recovery of the immobilized lipase was 1,716% and the specific activity increased to 77,460 U/g-protein, 17-fold higher than that of the free enzyme. The immobilized lipase displayed significantly enhanced thermostability and pH-resistance, and could efficiently catalyze transesterification to produce biodiesel at a conversion rate of 92.8%. Moreover, it possessed better recycling performance. After 20 cycles of repeated used, it still retained ca. 90% of its original activity, since the carbon nanotube−enzyme conjugates could be easily separated from the reaction mixture by using a magnet. This study provides a new perspective for biotechnological applications by adding a magnetic property to the unique intrinsic properties of nanotubes.

In light of the current global environment and focus on renewable energy sources, transesterification of oil feedstock catalyzed by lipase has been regarded as one of the most promising techniques for preparing biodiesel, a mixture of fatty acid alkyl esters (FAAEs). Although the lipase enzymatic reaction exhibits many advantages including broad adaptability to crude materials, mild reaction conditions, environmentally benign and ease of down-stream processing, reports of industrial applications have been very few^1^. The major reason is that the biocatalyst (lipase) itself is expensive and normally cannot be recycled, resulting in very high production costs. To date, various approaches have been adapted to try to circumvent the above shortcoming. Among them, immobilized lipase presents enormous potential for industrial application due to its enhanced tolerance to pH, heat, organic solvents and shear force, and because it is much easier to recover than the free form of the enzyme.

Hitherto, different sorts of materials, such as inorganic materials^2^, natural polymers^3^, and synthetic polymers^4^, have been used to immobilize enzymes and can improve their catalytic performance to some extent. In recent years, several types of nanostructured materials have been extensively employed including nanotubes, nanorods, nanorings, nanowires, etc.^5^,^6^. The merits of using these nanoscale structures for immobilization are that they reduce transfer resistance and maximize the functional surface area, increasing effective enzyme loading^7^. Carbon nanotubes (CNTs) in particular have attracted growing interest since their discovery in 1991^8^,^9^, mainly owing to their favorable physicochemical properties. In addition, CNTs can be easily functionalized and show great promise in various applications^10^,^11^,^12^. However, one bottleneck encountered when using CNT-immobilized enzymes is their recovery from the reaction mixture for repeated use. An effective solution is to endow the CNTs with a magnetic property^13^. Tan *et al*.^14^ successfully immobilized lipase on magnetic multi-walled carbon nanotubes (mMWCNTs) whose surfaces were loaded with magnetic iron oxide nanoparticles. And amyloglucosidase was immobilized on CNTs filled with magnetic iron oxide nanoparticles^15^. In both cases the immobilized enzymes displayed enhanced activities. Zhao *et al*.^16^ functionalized the magnetic CNTs with dendrimers to increase the number of active sites for enzyme immobilization on the CNTs and further improve enzyme loading and activity. Fan *et al*.^17^ also synthesized dendrimer-coated magnetic mMWCNTs for immobilization. They discovered that the concentrations of ionic iron used for preparing the magnetic CNTs affected both the saturation magnetization and the hydroxyl group content on the surface of the magnetic CNTs, where the former was inversely proportional to the latter, i.e., the amount of hydroxyl groups that could be used for modification decreased with increases in the saturation magnetization. In fact, the expected result is that the content of groups that can be modified should not be influenced by increases in saturation magnetization.

Thus, in this work, carbon nanotubes filled with magnetic iron oxide and further functionalized with polyamidoamine (PAMAM) dendrimers were first prepared and then used to immobilize lipase. The process is shown in Fig. 1 and the detailed procedures are described in the “Methods” section. Some specific characteristics of the immobilized lipase, including optimization of the immobilization conditions and the stability of the immobilized enzyme, were further examined. Moreover, the prepared immobilized lipase was utilized to catalyze transesterification for biodiesel production.

## Results

### Fabrication and characterization of MWCNTs and magnetic MWCNTs with immobilized *Burkholderia cepacia* lipase (BCL-mMWCNTs-G3)

**Figure 1:**
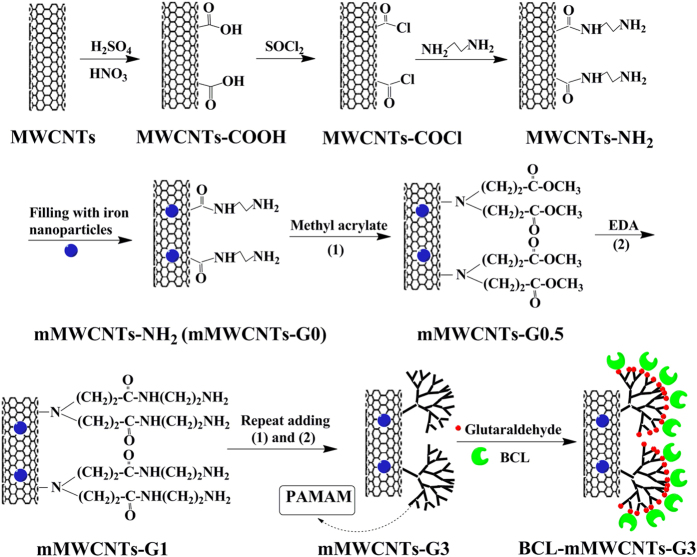
Schematic illustration of magnetic iron oxide nanoparticles filled carbon nanotubes modified with PAMAM dendrimers for BCL immobilization.

**Figure 2:**
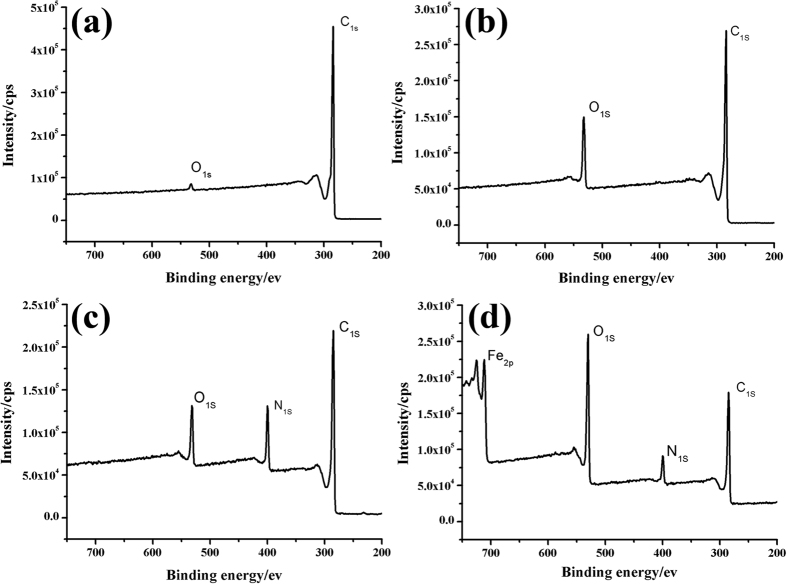
XPS spectra of the crude MWCNTs (**a**); oxidized MWCNTs (**b**); amino-functionalized MWNTs (**c**); amino-functionalized MWCNTs filled with magnetic iron oxide nanoparticles (**d**).

Figure 1 shows the synthesis strategy for mMWCNTs modified with PAMAM dendrimers (mMWCNTs-PAMAM). First, amino-functionalized MWCNTs (MWCNTs-NH_2_) were obtained. Then, the magnetic carbon nanotubes (mMWCNTs-NH_2_) were prepared through a facile approach that encapsulated iron oxide nanoparticles in the interiors of MWCNTs-NH_2_. As per the method of Zhao *et al*.^16^, the PAMAM dendrimers were grown on the surface of mMWCNTs up to the third generation to achieve mMWCNTs-G3, employing a divergent route starting from the mMWCNTs-NH_2_. A Michael addition reaction took place between the methyl acrylates and the pre-existing amino groups at a 2:1 mole ratio of propionate ester groups per amino group. Ester moieties subsequently reacted with ethylenediamine to complete the generation. The desired dendrimers were synthesized by repeating these two reactions. These processes were investigated with XPS (Fig. 2). XPS wide energy survey scans were used to characterize the surface composition of the MWCNTs. Two main peaks of C_1s_ and O_1s_ were present in all spectra. However, the O_1s_ signal varied due to improvement of the oxygen content of the treated MWCNTs compared to the crude MWCNTs. Meanwhile a peak corresponding to N_1s_ could be detected in the MWCNTs-NH_2_ and mMWCNTs-NH_2_ composite scans, confirming that MWCNTs-NH_2_ was successfully synthesized from the oxidized MWCNTs, and MWCNTs filled with iron oxide nanoparticles did not affect the surface amino-groups of MWCNTs-NH_2_. From Fig. 2d, Fe_2p_ peak demonstrated that MWCNTs containing iron were prepared and the iron was from iron oxide nanoparticles probably present in the inside or outside of the MWCNTs.

**Figure 3:**
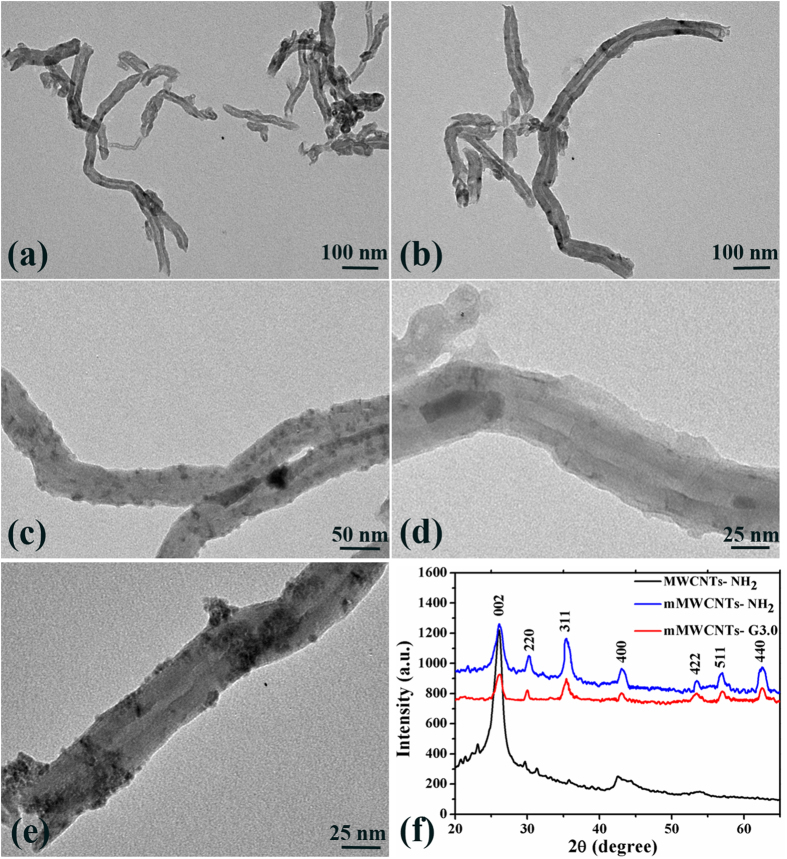
TEM images of oxidized MWCNTs (**a**); amino-functionalized MWCNTs (**b**); amino-functionalized MWCNTs with several Fe_3_O_4_ nanoparticles (**c**); enlarged image of a MWCNTs with Fe_3_O_4_ nanoparticles in the inner cavity (**d**); magnetic MWCNTs modified with PAMAM dendrimers (**e**); XRD patterns for MWCNTs (**f**).

TEM studies monitored the morphological changes of the MWCNTs and further confirmed successful encapsulation of iron oxide nanoparticles in the interiors of MWCNTs, as shown in Fig. 3. The TEM images of the oxidized MWCNTs and MWCNTs-NH_2_ before functionalization are devoid of any particle-like features (see Fig. 3a,b). After magnetic treatment, it can be seen that the pretreated MWCNTs are filled with Fe_3_O_4_ nanoparticles, while there are a few sporadic nanoparticles unspecifically adsorbed on the external walls of the MWCNTs (Fig. 3c,d). Figure 3e shows representative TEM images of the mMWCNTs-NH_2_ after modification with the PAMAM dendrimer. In Fig. 3e, the surface of the mMWCNTs-NH_2_ has become rough with, dendrimer molecules (G3) attached on the surfaces of the nanotubes, compared with mMWCNTs-NH_2_ without PAMAM dendrimer (Fig. 3c). XRD patterns (Fig. 3f) of the amino-functionalized MWCNTs contain two prominent signature peaks at 26.091 and 43.069. Other new peaks at 30.212, 35.421, 43.069, 53.469, 56.992 and 62.764 appeared in the XRD patterns of mMWCNTs-NH_2_ and mMWCNTs-G3, revealing a cubic iron oxide phase. These generated peaks are close to JCPD standards: Fe_3_O_4_, magnetite (89–3854, 2*θ* = 30.088, 35.439, 43.07, 53.432, 56.958 and 62.546)^18^.

**Figure 4:**
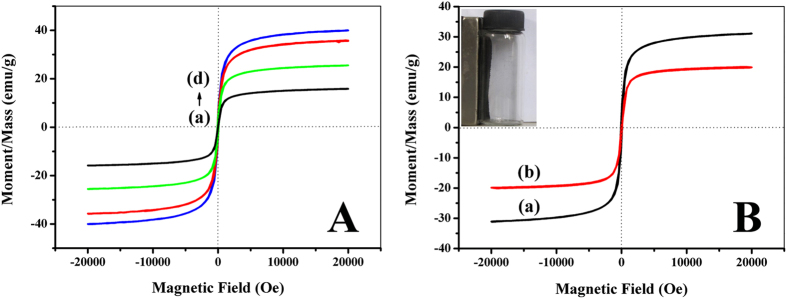
Magnetic hysteresis loops of magnetic MWCNTs hybrids (**A**): 0.0015 mol (a), 0.003 mol (b), 0.0045 mol (c), 0.006 mol (d); (**B**): mMWCNTs-G3 (a) and BCL-mMWCNTs-G3 (b). The insert shows BCL-mMWCNTs-G3 attached to the wall due to the attraction by the magnet.

The magnetic behaviors of the as-synthesized mMWCNTs-NH_2_ with different ferrous contents (0.0015, 0.003, 0.0045 and 0.006 mol), mMWCNTs-G3 and BCL-mMWCNTs-G3 were investigated using a SQUID analysis (Fig. 4). The magnetic hysteresis loops cross the zero point and display obvious S-like curves, indicating the characteristic superparamagnetic behavior of all nanoparticles. In Fig. 4A, the values of the saturation magnetization of the mMWCNTs-NH_2_ increase from 15.9 to 40.1 emu/g with increases in the metal ion concentration, which originates from the higher loading amounts of Fe_3_O_4_ nanoparticles in the interiors of MWCNTs. The nitrogen contents of mMWCNTs-NH_2_ prepared with different ferrous contents, meanwhile, were also investigated (Table 1), and showed the nitrogen content does not change with increasing ferrous ion concentration. These results indicated that the increase in saturation magnetization does not affect the content of amino groups that can be modified, which is exactly the desired outcome. Figure 4B shows the typical room temperature magnetization curves of mMWCNTs-G3 and BCL-mMWCNTs-G3. Their saturation magnetizations are 31.1 and 20.1 emu/g, respectively. The saturation magnetization of BCL-mMWCNTs-G3 is lower than that of mMWCNTs-G3 owing to the presence of enzyme-protein. The magnetization curves exhibit no hysteresis, indicating the superparamagnetic character of these samples. Generally, these properties allow for rapid separation of the immobilized enzyme from the reaction mixture using a magnet. Once the magnetic field has been removed, the immobilized enzyme can be easily dispersed by simple shaking.

**Figure 5:**
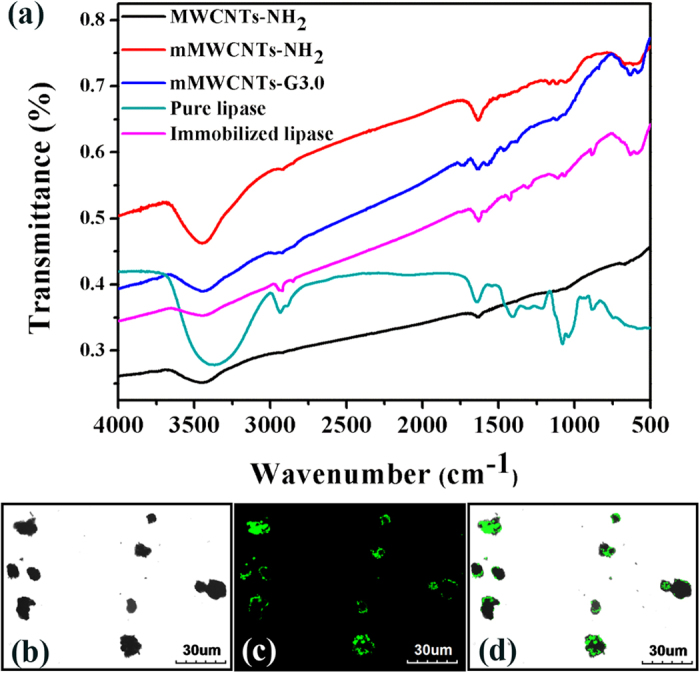
FT-IR spectra of MWCNTs-NH_2_, mMWCNTs-NH_2_, mMWCNTs-G3, pure lipase and immobilized lipase (**a**); confocal laser scanning microscopy image of the fluorescein *iso*-thiocyanate (FITC)-labeled BCL immobilized on mMWCNTs-G3 with a bright-field micrograph (**b**); FITC-labeled BCL fluorescence micrograph (**c**); overlay of a bright-field and FITC-labeled BCL fluorescence micrograph (**d**).

FT-IR spectra were further used to monitor the processes of mMWCNTs-G3 synthesis and lipase immobilization. In Fig. 5a, spectra of mMWCNTs-NH_2_, mMWCNTs-G3 and the immobilized lipase show characteristic absorptions at 580 cm^−1^ due to the Fe–O bond of magnetite compared with MWCNTs-NH_2_. Bands at 1635 and 1566 cm^−1^ are characteristic of amide (–CO–NH–) I and II bonds, respectively, indicating that the PAMAM dendrimer was bound to mMWCNTs. The peak of *ν* (C=O) of mMWCNTs-G3 corresponds to dendrimer-modified mMWCNTs-NH_2_ at 1739 cm^−1^, which shows that there indeed exist small amounts of –COO– groups in the nanocomposites. Elemental analysis was utilized to detect the surface nitrogen content of mMWCNTs-PAMAM, which gradually increased from mMWCNTs-G0 to mMWCNTs-G3 (Table 2). The nitrogen content, however, did not increase exponentially with the growth of the dendrimer, mostly due to steric interference during dendrimer growth on the mMWCNTs surface. After the immobilization of lipase on the mMWCNTs-G3 composites, the FT-IR spectrum of the immobilized lipase contained the characteristic bands of both lipase and the carrier. Additionally, confocal laser scanning microscopy (CLSM) analysis of BCL-mMWCNTs-G3 was performed to provide visual evidence, which directly confirmed the presence of the targeted BCL on the prepared mMWCNTs-G3 support. Before dyeing, the lipase was purified using the combined methods of ammonium sulfate fractionation, DEAE ion-exchange chromatography and gel filtration^19^. From the CLSM images portrayed in Fig. 5b–d, it can be clearly observed that the BCL-mMWCNTs-G3 resulted from immobilization of the fluorescein *iso*-thiocyanate (FITC)-labeled BCL onto the mMWCNTs-G3 support. In Fig. 5c, a strong green fluorescent signal of BCL is displayed in the fluorescence micrograph, and in Fig. 5d, the overlay of a bright-field and the FITC-labeled BCL fluorescence micrograph suggests the presence of FITC-labeled BCL on the mMWCNTs-G3 support after immobilization.

### Preparation of BCL-mMWCNTs-G3

The immobilization conditions, as is well known, have significant effects on the immobilization efficiency, activity recovery and specific activity of the immobilized lipase. The conditions, including glutaraldehyde concentration, pH value, lipase loading, coupling time and immobilization temperature, were all investigated in this study.

**Figure 6:**
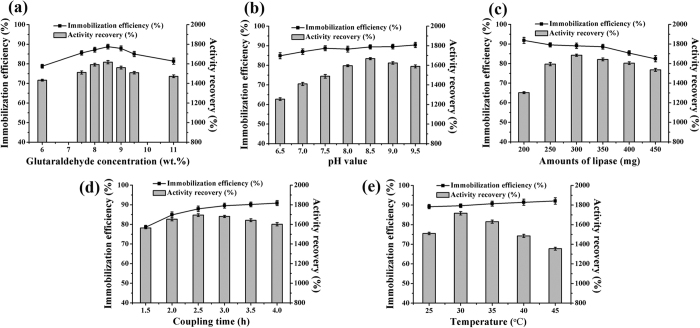
Effects of immobilization parameters on the immobilization efficiency and activity recovery. Glutaraldehyde concentration (**a**); pH value (**b**); amounts of lipase (**c**); coupling time (**d**); reaction temperature (**e**). The optimal immobilization conditions was glutaraldehyde concentration, 8.5 wt. %; pH value, 7.5; lipase amounts, 300 mg; coupling time, 2.5 h; reaction temperature, 30 °C.

Glutaraldehyde, a commonly used non-toxic cross-linker, constitutes a bridge connecting the carrier and enzyme. According to a previous study^17^, at low glutaraldehyde concentration, the amino groups of the carrier are inadequately activated, which results in insufficient cross-linking of the immobilized enzymes and the release of un-bound free enzyme into the aqueous medium. Too much glutaraldehyde causes intermolecular cross-linking of the enzyme molecules and the polymerization of glutaraldehyde. Therefore, a lower immobilization efficiency and activity recovery are obtained at both too low and too high concentrations. As shown in Fig. 6a, the glutaraldehyde concentration of 8.5 wt. % was most appropriate for cross-linking.

The pH value is also a critical factor in many reactions as well as in enzyme immobilization. As shown in Fig. 6b, an alkaline environment is beneficial for the Schiff base reaction between lipase and carrier. So, the immobilization efficiency was improved slightly at higher pH values. In addition, an acidic buffer seemed to be adverse to enzyme activity while basic conditions had a positive effect on the enzyme activity, with the highest value at pH 8.5.

In the range of 200 to 450 mg BCL, the maximal activity recovery was obtained at a lipase loading of 300 mg, and the immobilization efficiency decreased constantly at values higher or lower than 300 mg (Fig. 6c). Thus, a lipase amount of 300 mg was selected as the most appropriate for BCL immobilization.

In Fig. 6d, the immobilization efficiency and enzyme activity can be seen to gradually increase during the first 2.5 h. After that, the immobilization efficiency roughly leveled off. The enzyme activity, however, decreased for coupling times beyond 2.5 h, which was due to excess covalent binding between the aldehyde group of mMWCNTs-G3 and the amino group of BCL. As a result, 2.5 h was selected as the optimal coupling time for BCL immobilization.

As shown in Fig. 6e, temperature had little effect on the immobilization efficiency, which was always about 90%. In contrast, raising the temperature improved the enzyme activity and the highest value of 1,716% was observed at 30 °C. Beyond 30 °C, the activity recovery began to decrease. For this reason, 30 °C was considered the optimum temperature for BCL immobilization.

### Enzymatic properties of the immobilized BCL

**Figure 7:**
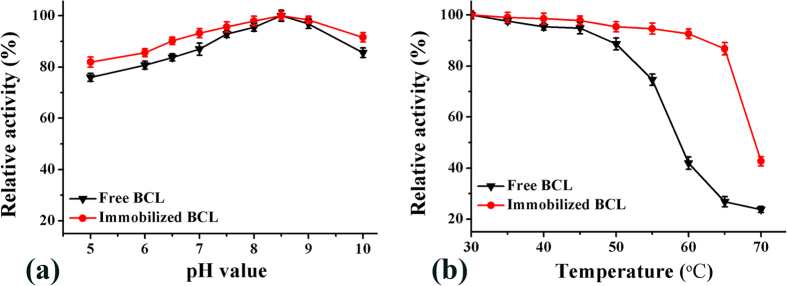
Stability of BCL-mMWCNTs-G3. pH stability (**a**) and Thermo-stability (**b**).

The stability of an enzyme is critical to its practical applications. To investigate the stability of the immobilized BCL, its activity was determined after incubation for 1 h at different pH values and temperatures. Figure 7a shows the pH stability of the free and immobilized BCL. As can be seen, the relative activity exhibited a similar trend, whereas the activity of the immobilized BCL decreased more slowly and to a lesser extent than free BCL. Moreover, BCL was more active at alkaline pH than that under acidic conditions. This result differed from that of Cao *et al*.^20^ who reported that PCL (*Pseudomonas cepacia* lipase, currently *Burkholderia cepacia* lipase, BCL) was more active at acidic pH, which might be due to differences in the lipase source and the properties of the carriers^21^. From Fig. 7a, it can be seen that immobilization significantly enhanced the pH stability of BCL. The relative activity of the immobilized BCL, when pH values ranged from 5.0 to 10.0, was above 80%. These results indicated that the conformation of BCL immobilized on the novel mMWCNTs-G3 was more stable, leading to a broad pH tolerance. The thermal stabilities of BCL and BCL-mMWCNTs-G3 were measured by incubation at different temperatures (30–70 °C). As shown in Fig. 7b, the relative activities of the free and immobilized BCL continuously decreased with increasing temperature. However, the decline in activity of the immobilized BCL was much lower than that of the free form. As can be seen, free BCL retained 88.6% of its initial activity after incubation for 1 h at 50 °C, while that of its immobilized counterpart was more than 95%, and even at 60 °C the residual activity remained over 90%. At 65 °C, the residual activity of free BCL was only 26.8%, while that of the immobilized BCL was still over 86%. This probably could be attributed to the carrier which strengthened the enzyme rigidity, thus protecting it from unfolding and obstructing the conformation transition of the enzyme at high temperature^22^. These results indicated that the temperature stability of BCL was considerably improved after the immobilization procedure.

### Enzymatic kinetic transesterification for biodiesel preparation

The catalytic activity of oxidized nanotubes and the as-prepared BCL-mMWCNTs-G3 in the transesterification reaction of soybean oil was detected. As expected, the oxidized nanotubes did not exhibit particular catalytic activities in the reaction at 1:5 oil/menthol molar ratios at 35 °C for 12 h. But after immobilization of BCL on mMWCNTs-G3, the catalysts were active and presented a high catalytic activity. Consequently, the catalyst activity is generated by loading the carrier with the lipase.

**Figure 8:**
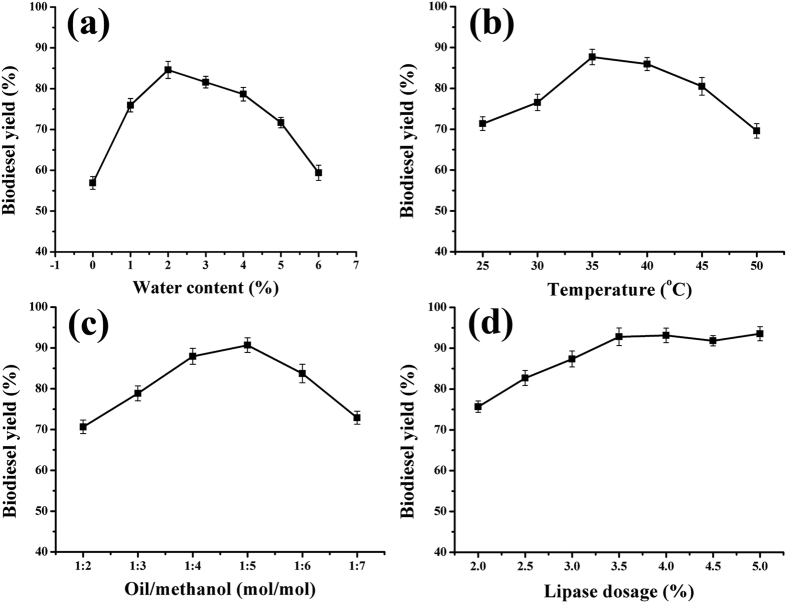
Effects of reaction parameters on biodiesel production catalyzed by BCL-mMWCNTs-G3. Water content (**a**); reaction temperature (**b**); oil to methanol molar ratio (**c**); lipase dosage (**d**).

To estimate the practical application of immobilized BCL, BCL-mMWCNTs-G3 was also investigated as an efficient nano-biocatalyst for enzymatic kinetic transesterification for producing biodiesel. A series of experiments were performed to determine the optimal conditions for fatty acid methyl ester (FAME) production (Fig. 8). As depicted in Fig. 8a, the yield of biodiesel increased markedly when 1% water was added to the reaction mixture; the highest biodiesel yield (84.6%) was obtained at 2%. Beyond 2%, the conversion rate began to decrease. As the esterification reaction is endothermic, an increase in temperature would be expected to increase the reaction rate^23^. Enzymes, however, exhibit the best catalytic activity in the appropriate temperature range for that protein, which also directly affects the reaction rate. Therefore, temperature is an important factor to be considered in the preparation of biodiesel. According to Fig. 8b, there is a rise in the biodiesel yield catalyzed by BCL-mMWCNTs-G3 when the temperature is gradually increased from 25 to 35 °C, but further increments in temperature to 50 °C caused a significant decline in the biodiesel yield. So, the optimum temperature for BCL-mMWCNTs-G3 was found to be 35 °C with biodiesel yields of about 87.6%. The influence of the molar ratio of oil to methanol on the biodiesel yield is shown in Fig. 8c. As the molar ratio increased from 1:2 to 1:5, the biodiesel conversion rate gradually increased to 90.7%, then began to decrease regularly when the amount of methanol was further increased. Thus, a molar ratio of oil to methanol of 1:5 is appropriate for FAME production. To reduce the production cost of biodiesel, the amount of BCL-mMWCNTs-G3 added to the reaction mixture was optimized. The maximal biodiesel yield was obtained at a lipase dosage of 4%, approximately 93.1% under the following conditions: moisture content 2% (based on the oil weight), molar ratio of oil to methanol 1:5, reaction duration 12 h, reaction temperature 35 °C, stirring rate 200 rpm and addition of methanol in three steps (Fig. 8d).

### Reusability of the immobilized enzyme

**Figure 9:**
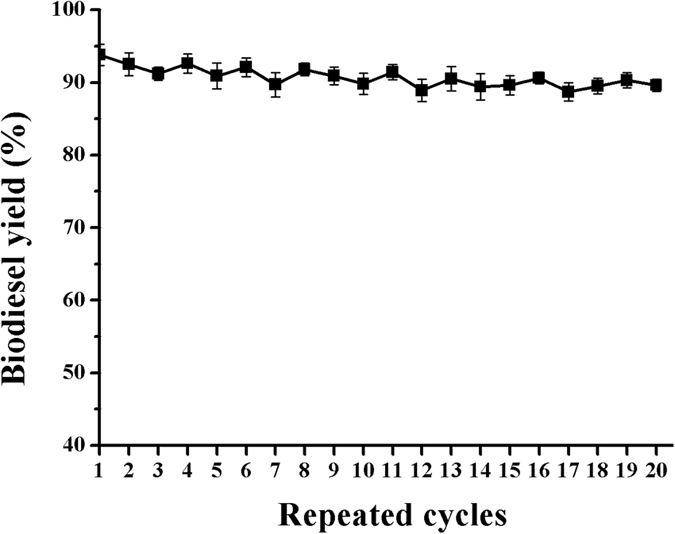
Reuse of BCL-mMWCNTs-G3 for biodiesel production.

One of objectives of using an immobilized enzyme is to design a more efficient biocatalyst that can easily be recovered and reused. To investigate the reusability of BCL immobilized on a functionalized mMWCNTs support, the immobilized enzyme was recovered by magnetic separation after each batch and washed with *t*-butanol to prepare it for the subsequent batch. The reusability of BCL-mMWCNTs-G3 is presented in Fig. 9. It was observed that BCL-mMWCNTs-G3 retained 89.6% yield after continually running for 20 cycles. Obviously, these results indicate that immobilizing the BCL significantly increases its operational stability.

## Discussion

In order to combine advantageous properties, such as ease of separation and recovery and sufficient active sites, into a single system for enzyme immobilization, in this study we synthesized dendrimer-functionalized carbon nanotubes filled with magnetic iron oxide.

CNTs are a kind of nanomaterial that has gained growing interest mainly because of their favorable properties such as a large specific surface area, greater affordability, unique electronic properties and small, hollow and layered structures^24^. However, the major drawbacks of CNTs are poor dispersibility in water or solvent and difficulty in separation after a catalytic reaction. Ke *et al*. and Ji *et al*.^25^,^26^ successfully immobilized lipases on oxidized CNTs via acid treatment, which not only solved the problem of dispersion but also achieved high immobilization efficiency and satisfying enzyme activities. Magnetism makes heterogeneous catalysis possible by which efficient separation of biocatalysts is made feasible. Chen *et al*.^27^ have synthesized magnetic CNTs by the impregnation method, with magnetic iron oxide nanoparticles loaded onto the surfaces of multi-walled carbon nanotubes. However, the ionic iron concentrations used for preparing magnetic CNTs influenced both the saturation magnetization and the amount of surface hydroxyl groups on the magnetic CNTs, and more interesting is that the former was inversely proportional to the latter^17^. In order to attain the properties of high saturation magnetization and sufficient active sites, in this work hyperbranched poly(amidoamine) (PAMAM) was grafted onto the outer surfaces of the magnetic carbon nanotubes filled with iron oxide. The results demonstrated that the pre-processed MWCNTs-NH_2_ is propitious for making the iron solution flow into the interiors by capillary force and encapsulating Fe_3_O_4_ nanoparticles in the interior of MWCNTs-NH_2_. After the grafting process, the surface of the mMWCNTs offered enough active sites for immobilization of the enzyme. The mMWCNTs-G3 displayed a fast response (30 s) to an external magnetic field, which indicated that this level of saturation magnetization would be adequate for the separation of the immobilized enzyme.

During processes of immobilization, the method of immobilization, cross-linker, lipase source and carrier are usually different, thus the immobilization conditions will change accordingly. In order to achieve high enzyme activities or covalent binding capacities of the immobilized lipase with glutaraldehyde as cross-linker, the factors affecting the immobilization conditions were explored. The results indicated that lipase loading, pH value and immobilization temperature were statistically significant to the activity recovery. Under the optimal conditions, the activity recovery of the immobilized BCL was as high as 1,716%, which was 17-fold higher than that of the free enzyme. The significant increase in enzyme activity is not only related to the carrier properties, but also to the unique characteristics of lipase and the immobilization method^17^,^28^. As is well known, the mechanism for improving the activity and stability of the immobilized lipase is extremely complicated. It is mainly attributable to a combination of the following factors. First, as mentioned above, dendrimer-modified nanomaterials can provide a larger surface area, resulting in higher immobilization efficiency and larger contact area between the substrate(s) and the enzyme, which would effectively decrease mass transfer resistance, leading to higher activity^26^. Second are the unique properties of lipase itself. The active centers of most lipases are covered by a so-called “lid” structure, which controls access of the substrate(s) to the active site. The secondary structure of the lipase would probably change during immobilization, and the “lid” might be opened to some extent for the substrate(s), which would provide an easier access, leading to an increase in lipase activity^29^. The third factor is related to the immobilization method. In this work, a Schiff base reaction occurred between ε-NH_2_ groups on the surface of BCL and mMWCNTs-G3 activated by glutaraldehyde (mMWCNTs-G3-GA) via a covalent method. The 3D structure of BCL was analyzed and the distribution of ε-NH_2_ groups (lys residues) is shown in Figure S1. It can be seen from Figure S1 that the lys-residues are far from the catalytic site, so they are suitable for selection as attachment points to covalently combine on the surface of the carrier. Consequently, very little damage was done to the lipase activity, that is to say, this technique retained nearly all of the activity of the lipase once immobilized. This phenomenon was also observed in a previous study^30^. In contrast, conventional immobilization technologies immobilize the enzyme randomly with some of the enzyme catalytic active centers attached tightly to the surface of the matrix, resulting in some extent of enzyme activity loss. Actually, the activity of the product of oriented-immobilization was enhanced about 10-fold compared to that of a conventional immobilization^31^.

Immobilized lipase was used to catalyze transesterification for biodiesel preparation. During the process of transesterification, the biodiesel yield can be affected by a variety of reaction parameters. In transesterifications catalyzed by most of the lipases, water is essential to the reaction system^32^. There are two major explanations. One is that lipase displays the unique feature of acting as the interface between the organic phase and the aqueous phase, so the lipase activity generally depends on the interfacial area^33^. The other is that the reaction mechanism of an enzyme-catalyzed alcoholysis reaction in a micro-aqueous phase is composed of multiple sequential hydrolysis and esterification processes: i.e., triglyceride is hydrolyzed first into diacylglycerol and free fatty acid (FFAs); then the esterification reaction occurs between fatty acid and short chain alcohols through catalysis by lipases; next, diacylglycerol is continually hydrolyzed into monoacylglycerol and FFAs, and the esterification reaction reoccurs. Hydrolysis and esterification reactions occur in turn until the glycerides are completely hydrolyzed into glycerol and the generated FFAs are completely esterified into fatty acid alkyl esters (biodiesel)^34^. However, the moisture concentration can have positive or/and negative effects on the catalytic activity of lipase. Too much water in the reaction mixture might make the lipase more flexible, and moreover water is a reaction product in the biodiesel preparation process, which will lead to some unintended side reactions such as hydrolysis. For example, Royon *et al*.^35^ reported that commercial Novozym 435 lipase showed high catalytic activity in transesterification with no extra water added to the reaction system. Therefore, the amount of moisture added should depend on the lipase type, feedstock oil, organic solvent, and immobilized support.

Similarly, alcohols play dual roles in the alcoholysis reaction. They serve as reaction substrates taking part in biodiesel production, thus tending to push the reaction process in the direction of synthesis; at the same time, they are harmful to proteins in excessive proportions. Therefore, we not only chose an appropriate strategy for adding alcohol, but also added the appropriate amounts of alcohol to the reaction mixture in order to minimize damage to the enzyme and ensure the maximum reaction rate. In this study, three-step addition of methanol was employed with time intervals of 4 h between each step based on previous research^36^; the optimal amount of alcohol added to the reaction was also determined (Fig. 8c).

As is well known, immobilization is a good way to enhance the lipase stability and allow for it to be recycled. Conventional immobilization techniques include physical adsorption and covalent bonding. Among immobilization methods, covalent bonding connects the enzyme and carrier molecules firmly, so that lipases are not easily washed away. Although this binding process is more complex and may reduce enzyme activity compared to the native free enzyme, these disadvantages can be compensated by the recycle ability of the immobilized enzyme, hence defraying biodiesel production costs in the long term. However, the biodiesel yield would be expected to decrease slightly with increasing numbers of cycles owing to the negative effects of polar solvents, by-products of glycerol and mechanical damage of rotation to the immobilized lipase. In conclusion, the transesterification reaction catalyzed by BCL-mMWCNTs-G3 was promising not only because of the batch reaction but also because of the consecutive reactions.

Generally speaking, we successfully prepared biocompatible mMWCNTs-G3 and developed an easily separable immobilized enzyme system using this carrier. Enzyme recycling using magnetic separation proved to be an efficient means of recovering the coated enzyme. The obtained BCL-mMWCNTs-G3 had improved pH and temperature adaptability, higher activity and greater stability by comparison with its free counterpart. Furthermore, the immobilized BCL was used for biodiesel preparation, and demonstrated high conversion yields and better operational stability. This study demonstrated that mMWCNTs-G3 could be considered a good support for immobilization of an enzyme, and the as-prepared BCL-mMWCNTs-G3 is a promising nano-biocatalyst for biocatalytic reactions. Future studies will include the concurrent immobilization of two lipases or lipase with other enzymes on functionalized CNTs to increase biofuel production, which will illustrate the advantages of using CNTs for catalytic aims in parallel with other weaker durable nanomaterials.

## Methods

### Chemicals

*Burkholderia cepacia* lipase (BCL) powder was purchased from Amano Enzyme Inc. (Nagoya, Japan) and used without further purification. Multi-walled carbon nanotubes (40–60 nm diameter, purity >95%) were obtained from Nanotech Port Co. Ltd. (Shenzhen, China). The reference standards of fatty acid methyl esters were commercially obtained from Sigma-Aldrich. All other reagents such as glutaraldehyde (GA), ammonium iron (II) sulfate hexahydrate, methyl acrylate, ethylenediamine (EDA) and thionyl chloride were of analytical grade and purchased from Sinopharm Chemical Reagent Co. Ltd. (Shanghai, China).

### Analysis of the 3D-structure of BCL

The 3D structural model of BCL (pdb identifiers: 3LIP) obtained from the NCBI (http://www.ncbi.nlm.nih.gov/) was employed to analyze the surface-exposed amino groups using PyMOL (2.7.6)^37^.

### Preparation of magnetic MWCNTs (mMWCNTs)

In this process, amino-functionalized MWCNTs (MWCNTs-NH_2_) were firstly prepared via a simple and effective method as reported by Pan *et al*.^38^. MWCNTs-NH_2_ were filled with Fe_3_O_4_ using the method of Goh *et al*. with a slight modification^15^. Some ammonium iron (II) sulfate hexahydrate was dissolved in 20 mL of a dark green solution of deionized (DI) water and hydrazine hydrate in a volume ratio of 3:1. MWCNTs-NH_2_ (0.25 g) was added to the solution. The mixture was then sonicated for 30–60 min to disperse it evenly and the pH value was adjusted to 11–13 using ammonia solution under the condition of intense mechanical stirring that continued for 24 h to facilitate the uptake of the iron solution into the MWCNTs. Then the above mixture was refluxed at 130 °C for 2 h to produce solid Fe_3_O_4_ nanoparticles in the MWCNTs-NH_2_ and filtered and washed with N, N’-dimethylformamide, ethanol and finally DI water. To remove any Fe_3_O_4_ nanoparticles on the surface of the MWCNTs-NH_2_, they were washed with dilute hydrochloric acid and then rinsed with DI water, and dried at 60 °C under vacuum overnight to obtain magnetic MWCNTs-NH_2_ (mMWCNTs-NH_2_, namely mMWCNTs-G0), as shown in Fig. 1.

### Growth of PAMAM dendrimers on the surface of MWCNTs initiated by mMWCNTs-NH_2_ (mMWCNTs-PAMAM)

As-prepared mMWCNTs-NH_2_ initiator (1.0 g) was sonicated for 1 h in 20 mL dry methanol. Simultaneously, methyl acrylate (20 mL) and dry methanol (80 mL) were mixed and stirred in an ice-water bath for 30 min, and carefully dropped into the above mixture, with continuous stirring. The reaction was conducted at room temperature for 24 h, and was flushed with N_2_ throughout the whole process. Next, the product was collected using a magnet and washed with methanol five times to remove unbound polymer or free reagents, and dried at 60 °C under vacuum overnight. The obtained sample was defined as mMWCNTs-G0.5. Then, mMWCNTs-G0.5 (1.0 g) and dry methanol solution (20 mL) were mixed and sonicated for 1 h. Meanwhile, anhydrous ethylenediamine (20 mL) and dry methanol solution (80 mL) were blended and agitated in an ice-water bath for 30 min, and then added dropwise to the above mixture. The reaction was performed at room temperature for 24 h under nitrogen. The product was then collected magnetically and washed with methanol five times, and dried at 60 °C under vacuum overnight. The obtained product was defined as mMWCNTs-G1. Stepwise growth using methyl acrylate and ethylenediamine was repeated until the desired number of generations (up to generation 3) on the surface of mMWCNTs-NH_2_ appeared (Fig. 1). Additionally, according to the literature^16^,^39^, the amount of enzyme bound increased quickly as the generation of dendrimers grew from G0 to G3, then slowed down with further increases in generations. Therefore, considering the cost and time required to synthesize the composites, mMWCNTs-G3 was used for all experiments.

### Covalent immobilization of lipase on mMWCNTs-G3

An appropriate amount of mMWCNTs-G3 composites was dispersed in ethanol, followed by adding 25% glutaraldehyde (GA) and shaking in a thermostatic shaker at 200 rpm at 30 °C for 10 h. The product was isolated by magnetic separation and washed several times with DI water to remove excess GA. The obtained sample was defined as mMWCNTs-G3-GA. Then, 0.1 g mMWCNTs-G3-GA was dispersed in 5.0 mL phosphate-buffered solution (PBS, pH 7.0, 0.05 M), and some lipase was added and sonicated to re-disperse. The mixture was shaken at 200 rpm at a given temperature and time, and thoroughly separated magnetically and rinsed with fresh buffer to remove unbound and nonspecifically adsorbed lipase.

### Determination of esterification activity

The activities of the immobilized and free lipases were assayed by the esterification of lauric acid and 1-dodecanol^40^: a certain amount of the immobilized or free lipase was added to a mixture (10 mL) consisting of lauric acid (0.2 M) and 1-dodecanol (0.2 M) in isooctane with a small amount of water (0.01 mL), and the reactions were carried out at 30 °C for 30 min with continuous shaking at 200 rpm. A 1 mL sample was withdrawn and mixed with 15 mL of ethanol-acetone (1:1, *v/v*) to stop the reaction. The remaining acid in the sample was measured by titration with 0.05 M NaOH solution. One unit of enzyme activity (U) was defined as the amount of lipase that consumed 1μmol of lauric acid per minute under the assay conditions. The amount of immobilized enzyme was determined according to the method described by Bradford with bovine serum albumin (BSA) as the standard^41^. The immobilization efficiency (%) and activity recovery (%) were calculated via Eqs (1),(2),(3) 42.


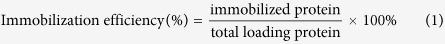



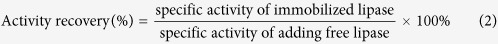



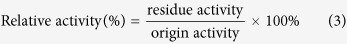


### Measurements and characterization

MWCNTs samples were visualized using transmission electron microscopy (TEM) with a Hitachi H-7000FA microscope. TEM specimens were prepared by placing a drop of the sample suspension on 230 mesh copper grids coated with carbon. The magnetic properties were examined using a superconducting quantum interference device (SQUID, Quantum Design, USA) at room temperature. X-ray diffraction (XRD) was carried out using an X’ Pert PRO X-ray diffractometer with Ni-filtered Cu Ka radiation (PAN analytical B.V., Almelo, Netherlands). The FT-IR experiments were performed in transmission mode using the KBr pellet technique (Bruker, VERTEX 70, Germany). An elemental analyzer was used to determine the nitrogen content in each generation of the mMWCNTs-PAMAM nanocomposites (Elementar Co., Vario Micro cube, Germany). Surface chemistry was investigated by X-ray photoelectron spectroscopy (XPS) using a Kratos Axis Ultra instrument equipped with a monochromated Al Kα X-ray source (Kratos Co., AXIS-ULTRA DLD-600W, Shimadu). BCL-mMWCNTs-G3 was visualized via confocal laser scanning microscopy (CLSM) with a SIM scanner (Olympus FV1000 Co., Japan) to determine the fluorescence signal from the fluorescein iso-thiocyanate (FITC)-labeled BCL after immobilization on the mMWCNTs-G3 support. The targeted BCL was labeled with FITC as follows: BCL was dissolved in PBS (pH 7.0, 0.05 M) at a final concentration of 10 mg/mL; FITC was dissolved in dimethyl sulfoxide (DMSO) at a final concentration of fluorescein dye of 1 mg/mL. BCL solution (5 mL) and FITC (100 μL) were mixed for 24 h at 4 °C in the absence of light, and then the residual FITC was removed from the mixture via extensive dialysis with DI water.

### Enzymatic-catalyzed biodiesel production

The enzymatic transesterification reaction was conducted in a solvent-free system. The reactions catalyzed by immobilized BCL were conducted in a stoppered 50 mL shake flask at a stirring rate of 200 rpm. The reaction mixture was composed of soybean oil (2.19 g), methanol, immobilized BCL (77,460 U/g-protein) and some water. The influences of crucial operating parameters on the biodiesel yield were systematically investigated, such as the immobilized lipase dosage, molar ratio of alcohol to oil, water content and reaction temperature. The methanol was added in three steps at 4 h intervals. All dosage percentages were based on the oil weight, unless otherwise stated. The lipases in the reaction mixture were recycled using a magnet, washed with *t*-butanol and then added to a fresh batch of reaction medium to examine the reusability of the immobilized lipase. Samples were collected from the reaction mixture after a certain reaction period, and then centrifuged at 13800×g for 5 min to obtain the supernatant. Supernatant (10 μL), 300 μL of 1.0 mg/mL heptadecanoic acid methyl ester (as internal standard) and 290 μL hexane were mixed thoroughly for gas chromatography (GC) analysis to determine the fatty acid methyl ester (FAME, namely biodiesel) yield.

### GC determination

The method of GC determination was reported in our previous studies^43^. The biodiesel yield was determined using a GC-9790 gas chromatograph system (Agilent HP-INNOWAX capillary column 30 m × 0.25 mm × 0.25 μm, J&W Scientific, Folsom, CA, USA). The above-mentioned mixed sample (1.0 μL) was injected into the GC, and the column initial temperature was 180 °C and increased to 230 °C at a rate of 3 °C/min and then maintained at 230 °C for 3 min. The injector and detector temperatures were set at 230 °C and 280 °C, respectively. The biodiesel yield (%) was defined as the conversion of oil and calculated as the total FAME content in the converted oil sample. The formula for this calculation is similar to that previously reported in the literature^43^.

### Experimental design and statistical analysis

All experiments were conducted in three parallel replicates. The data were analyzed using SAS 9.0 software (SAS Institute Inc., Cary, NC, USA). Analytical data were expressed as the average value ± standard deviation, and the graphs were plotted using Origin 8.0 software (Origin Lab Co., Northampton, MA, USA).

## Additional Information

**How to cite this article**: Fan, Y. *et al*. Carbon nanotube filled with magnetic iron oxide and modified with polyamidoamine dendrimers for immobilizing lipase toward application in biodiesel production. *Sci. Rep.* **7**, 45643; doi: 10.1038/srep45643 (2017).

**Publisher's note:** Springer Nature remains neutral with regard to jurisdictional claims in published maps and institutional affiliations.

## Supplementary Material

Supporting Information

## Figures and Tables

**Table 1:** The nitrogen content of mMWCNTs-NH_2_ with different ferrous content.

Ferrous content (mol)	Nitrogen content (%)
0	2.59 ± 0.02
0.0015	2.61 ± 0.01
0.003	2.53 ± 0.03
0.0045	2.49 ± 0.03
0.006	2.55 ± 0.02

**Table 2:** The nitrogen content of each generation of mMWCNTs-PAMAM.

Generation	Nitrogen content (%)
G0	2.58 ± 0.01
G1	3.86 ± 0.02
G2	5.56 ± 0.03
G3	6.47 ± 0.03
