# Supplementary material for: Carbon nanotube filled with magnetic iron oxide and modified with polyamidoamine dendrimers for immobilizing lipase toward application in biodiesel production
Source: Sci Rep. 2017 Mar 30;7:45643. doi: 10.1038/srep45643 (PMC5372472; doi:10.1038/srep45643)

**Supporting Information**

**Carbon nanotube filled with magnetic iron oxide and modified with** **polyamidoamine dendrimers for immobilizing lipase toward application in biodiesel production**

Yanli Fan, Feng Su, Kai Li, Caixia Ke, Yunjun Yan^*^

Key Laboratory of Molecular Biophysics of the Ministry of Education, College of Life Science and Technology, Huazhong University of Science and Technology, Wuhan 430074, P. R.China

^*^**Corresponding author: Yunjun Yan**

**Phone/Fax:** +86-27-87792213;

**E-mail:** [yanyunjun@hust.edu.cn](mailto:yanyunjun@hust.edu.cn)

**Supplementary Figure Captions**

Fig. S1 The distribution of amino groups in BCL molecules (Lys-residues were dyed yellow while red and blue regions represent catalytic active sites and oxyanion hole, respectively).

**Fig. S1**


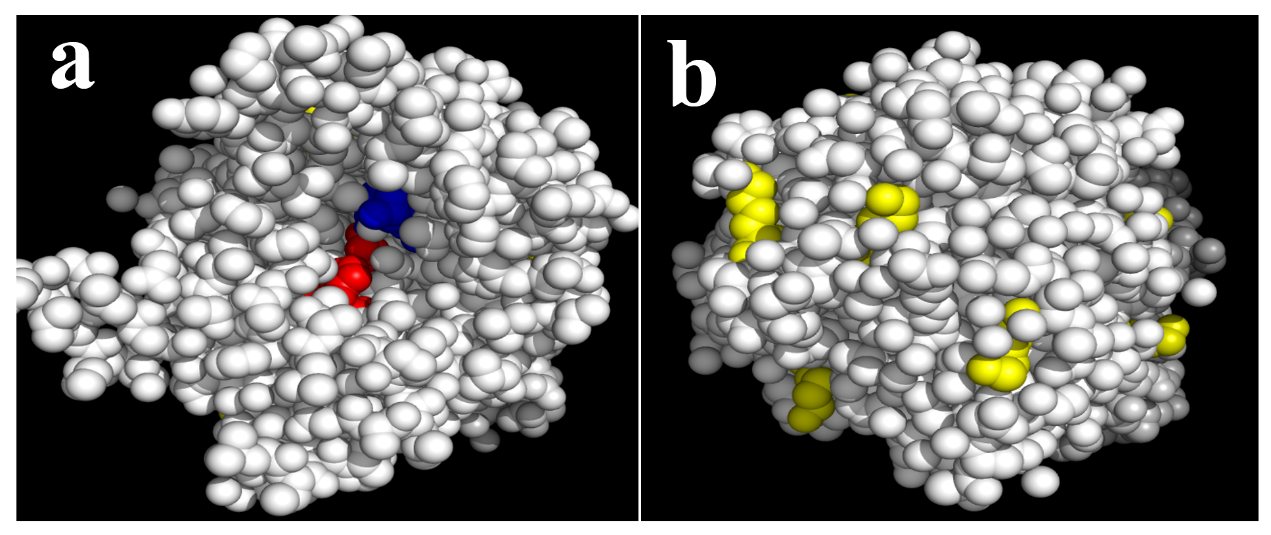

Supplement: Supporting Information [file srep45643-s1.docx]
